# Unexpected success in early post-transplantation renal vein thrombosis: A case report and literature review 

**DOI:** 10.5414/CNCS110407

**Published:** 2021-02-19

**Authors:** Joana Eugénio Santos, Ana Gaspar, Sara Querido, Cristina Jorge, André Weigert, Henrique Mesquita Gabriel, António Martinho, Domingos Machado

**Affiliations:** 1 Division of Nephrology, Hospital Espírito Santo de Évora, Évora,; 2 Division of Nephrology, Hospital Prof. Dr. Fernando da Fonseca, Amadora,; 3 Division of Nephrology,; 4 Division of Cardiology, and; 5 Division of Surgery, Hospital de Santa Cruz, Carnaxide, Portugal

**Keywords:** renal vein thrombosis, kidney transplantation, early postoperative period, vascular complications, deep vein thrombosis, balloon catheter, aspiration venous thrombectomy

## Abstract

Background: Allograft renal vein thrombosis can cause graft loss during the early postoperative period. This diagnosis is sometimes elusive, requiring a strong suspicion. On the other hand, several authors have recognized risk factors for allograft renal vein thrombosis, but neither a preventive approach nor a treatment have been recommended for this complication. Case presentation: We present a case report of early allograft renal vein thrombosis, preceded by femoral common deep vein thrombosis in a recipient of a third kidney transplant. Despite femoral common deep vein thrombosis treatment with low-molecular-weight heparin and progressive improvement of renal function to a nadir serum creatinine of 0.51 mg/dL, the patient experienced a sudden episode of anuria on postoperative day 5. Doppler ultrasonography strongly suggested the diagnosis of allograft renal vein thrombosis. The patient underwent balloon catheter and aspiration venous thrombectomy, followed by unfractionated heparin perfusion. After 4 days of anuria and multiple blood transfusions, when allograft nephrectomy was contemplated, diuresis suddenly resumed. After 1 year of follow-up, the patient still has a normal renal function. Conclusion: This case report shows successful treatment of allograft renal vein thrombosis associated with deep vein thrombosis in the first week of transplantation, using balloon catheter and aspiration venous thrombectomy followed by perfusion of unfractionated heparin. The authors suggest this technique as a treatment option for transplant renal vein thrombosis. However, they reinforce the importance of individualized treatment and they remind that a delay may jeopardize the potential benefit of the procedure.

## Introduction 

Allograft renal vein thrombosis (ARVT) is one of the leading causes of early graft dysfunction, with a reported prevalence of 0.1 – 4.2% of all transplants [1] and it remains a significant cause of graft failure and nephrectomy during the early postoperative period [2, 3]. 

Identified risk factors include: anatomical peculiarities, namely multiple veins, wide disparities in vessel size, and extrinsic compression by lymphocele or hematoma, and surgical complications such as renal vein kinking, anastomotic defect, and endothelial injury during the procedure. Other known predictive risk factors for the development of ARVT include chronic prothrombotic state, lengthened ischemic time, deep venous thrombosis, and acute rejection [1, 4, 5, 6]. Prevention is not always possible. Therefore, it is essential to establish the most suitable treatment when ARVT is detected. 

Recently, successful results with surgical and percutaneous thrombectomy in combination with anticoagulant or thrombolytic therapy were reported [7, 8]. However, no randomized controlled trials (RCTs) assessed the efficacy of different treatments, and these reports failed to provide renal allograft outcomes in the short and long term. 

The purpose of this article is to support balloon catheter and aspiration venous thrombectomy coupled with anticoagulation as a successful treatment for ARVT during the early post-transplantation period. 

## Case presentation 

A 52-year-old Caucasian female with end-stage renal disease (ESRD) of unknown etiology since the age of 12 presented to our hospital for her third kidney transplant (KT) in March 2019. Her first KT in 1979, from a living related donor, was complicated by severe acute rejection and consequently need of nephrectomy on postoperative day 5. In 1988, she received a second KT, from a deceased donor. It failed after 27 years due to chronic renal allograft dysfunction, and she returned to regular hemodialysis (HD) after creation of a radio-cephalic fistula in 2015. Her past medical history included a bilateral oophorectomy in 1994, cholecystectomy for gallstone in 2002, and hypertension secondary to chronic renal disease. She did not have any previous history of arterial or venous thrombosis or any HD access thrombosis before the transplant. The patient received a KT from a deceased male donor whose cause of death was cerebral hemorrhage. At KT time the complement-dependent cytotoxicity (CDC) crossmatch, T cell flow cytometry crossmatch (FCW) and donor-specific antibody (DSA) evaluated by single antigen test, were all negative. Donor was cytomegalovirus immunoglobulin-G negative, while recipient was positive. The total cold ischemia time was 12 hours 45 minutes. The right kidney was transplanted into the right iliac fossa. The donor renal artery was anastomosed to the recipient’s right external iliac artery, and the donor renal vein, together with a patch of donor vena cava, was anastomosed to the recipient’s right external iliac vein, both end-to-side. Lastly, the graft ureter was anastomosed to the urinary bladder of the recipient. 

Induction immunosuppression included polyclonal antibody sera (Thymoglobulin, Genzyme, Amsterdam, Netherlands) followed by triple immunosuppressive therapy with tacrolimus, mycophenolate mofetil, and prednisolone. Immediate kidney reperfusion and urinary output were observed during the surgery. The early postoperative course was unremarkable with Doppler ultrasonography (US) of the allograft in the 1^st^ postoperative day unveiling good global vascularization and resistive index (RI) of 0.71 – 0.77. 

**Figure 1.**
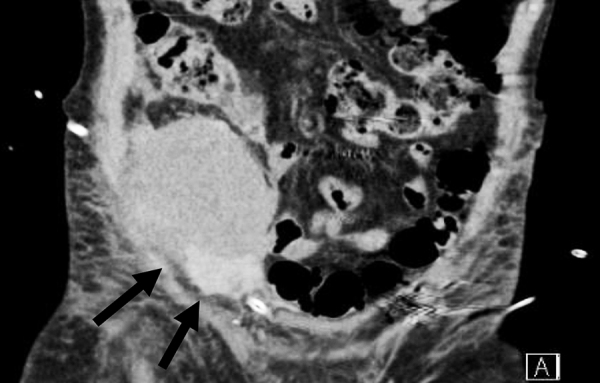
Pelvic computed tomography showing peri-graft hematoma with large diameter of 13 cm (black arrow).

In the 2^nd^ postoperative day, the patient presented a significant decrease in hemoglobin level (Hb 9 to Hb 5 g/dL) accompanied by symptomatic hypotension. Doppler US and pelvic computed tomography scan revealed a peri-graft blood collection with a diameter of 13 cm, with no other abnormalities (Figure 1). The patient was managed with conservative treatment, receiving two blood transfusions. Several hours later, the patient developed right proximal leg swelling accompanied by mild pain. Although hourly urine output remained stable, the affected thigh was much more swollen and a deep vein thrombosis (DVT) was suspected in the morning. After the diagnosis of extensive femoral common vein thrombosis, systemic anticoagulation was initiated using low-molecular-weight heparin (1 mg/kg/day). Laboratory evaluation for thrombophilic states including activated protein C resistance, functional protein C, functional protein S, functional anti-thrombin III, homocysteine, and lupus anticoagulant did not reveal any abnormalities. 

**Figure 2.**
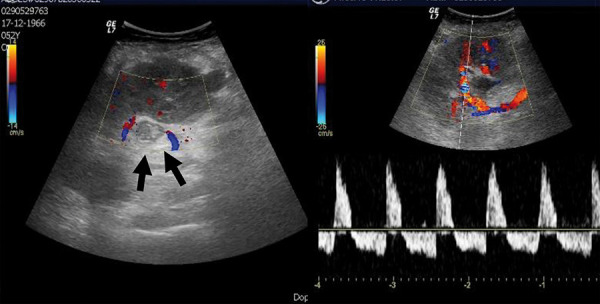
Initial Doppler ultrasonography showing left enlarged renal vein filled with hypoechogenic content, without Doppler signal (black arrow), and on****right arterial reversed diastolic flow.

Despite this complication, she had a progressive improvement of the renal function reaching a nadir of serum creatinine (sCr) 0.51 mg/dL. However, on postoperative day 5, sudden anuria established. Urgent Doppler US distinguished an enlarged renal vein filled with hypoechogenic content, without Doppler signal and an arterial reversed diastolic flow suggesting ARVT (Figure 2). The patient was immediately submitted to balloon catheter and aspiration venous thrombectomy, which was initially attempted through the right femoral vein, without guide-wire progression due to an organized thrombus. So, it was after what performed through the left femoral vein, documenting a DVT on the right external iliac vein which was probably caused by stenosis on the previous site of renal graft venous anastomosis. Initially, 2,500 IU of heparinized saline were infused, followed by balloon angioplasty until 10 atm, aspiration venous thrombectomy, and a new infusion of 5,000 IU of heparinized saline. Final venography showed patency of the left external iliac vein without filling of the graft’s renal vein. Continued perfusion of unfractionated heparin to maintain an activated partial thromboplastin time test (aPTT) of 70 – 110 seconds followed this procedure. 

**Figure 3.**
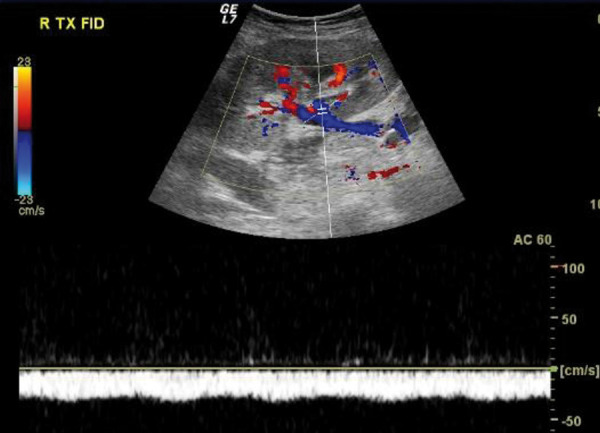
Doppler ultrasonography on day 5 after balloon catheter and aspiration venous thrombectomy showing Doppler signal on the renal vein with continued diastolic flow.

On the 1^st^ day after balloon catheter and aspiration venous thrombectomy, the allograft Doppler US still showed features of renal vein thrombosis, despite signals of mild partial re-permeabilization of the right common femoral and external iliac vein. After the procedure, the patient experienced recurrent hemoglobin drop and multiple hematomas, requiring additional blood transfusions. HD had to be resumed due to hypervolemia and blood urea nitrogen retention (sCr 4.1 mg/dL and urea 105 mg/dL). On the 4^th^ day post procedure, the patient remained anuric. Renal allograft nephrectomy was considered, given the hemorrhagic risks of maintaining anticoagulation and the unsuccessful results achieved so far. However, on this day, a new allograft Doppler US showed a short stretch of a segmental external branch of the renal vein with a weak Doppler signal despite extensive signs of renal vein thrombosis. These encouraging findings led the transplantation team to a “wait and see” approach. On the next day, urinary output was recovered and Doppler US confirmed re-permeabilization of the renal vein and segmental branches (Figure 3). 

Unfractionated heparin perfusion was maintained for 13 days after balloon catheter and aspiration venous thrombectomy; subsequently, warfarin was used. The renal function gradually improved and reached a SCr of 0.66 mg/dL 26 days after the transplantation procedure. After 1 year of follow-up, the patient maintains long-term anticoagulation with warfarin, the same maintenance immunosuppressive therapy with tacrolimus, mycophenolate mofetil and prednisolone and normal renal function with a SCr of 0.7 mg/dL (eGFR using CKD-EPI 100.1 mL/min/1.73m^2^) without any other complications. 

## Discussion 

Recently, the improvements in the management of medical complications of kidney transplantation have improved graft survival in the first year after transplantation. However, the incidence of surgical complications still amounts to 15.9%, including a 2.7% incidence rate of vascular thrombosis [9]. 

In the present case, the diagnosis of DVT prompted anticoagulant therapy. However, and despite this reasonable treatment, the patient developed ARVT likely by DVT extension. Nonetheless, our patient had some ARVT risk factors, such as a right kidney graft, immunosuppression with MMF, and possible external compression by a peri-graft hematoma. 

DVT in a kidney transplant recipient is rare but can cause allograft loss, rupture, and death in addition to the general risk of pulmonary thromboembolism [10, 11]. Similarly, ARVT frequently causes allograft loss. This vascular complication mainly occurs in the first 2 weeks after transplantation. Hypercoagulability state is a risk factor for both conditions and it is frequent in the first month after transplantation, as a consequence of postoperative period and end-stage renal disease [12, 13, 14]. It is a modifiable factor since prophylactic anticoagulant or antiplatelet therapy can reduce the risks of these complications. However, effective management of thrombophilic states in this period presents an ongoing challenge and remains controversial, as hematuria and perirenal bleeding are frequent post kidney transplantation. 

Because of the low incidence of DVT and ARVT (1.7%) and hemorrhagic risks, many authors do not suggest the use of chemical prophylaxis in the first month after transplantation [15]. Nevertheless, some authors suggest postoperative management of the patients with a hypercoagulability profile or technical issues with low-dose aspirin or heparin [5, 6, 16]. 

There are studies showing a decreased incidence of ARVT with a low dose of aspirin without hemorrhagic complications [17, 18]. Similarly, prophylaxis with low-molecular-weight heparin seems to be an effective and safe method [16]. Finally, studies showing that the optimal aPTT ratio during unfractionated heparin prophylactic use, to prevent thrombosis and limit bleeding risk, appeared to be 1.5 – 1.9 [19]. 

As ARVT prevention is not always possible in addition to the doubtful role of anticoagulant or antiplatelet prophylaxis, it is essential to establish the most suitable treatment [20]. 

In the described case, renal vein thrombosis was successfully treated with balloon angioplasty and manual aspiration of clots, followed by unfractionated heparin perfusion for 13 days. Surgical exploration with venous thrombectomy could also have been suitable for our patient, as she had a previously identified hematoma. However, this strategy was not chosen because of simultaneous femoral common vein thrombosis without apparent technical cause. On the other hand, percutaneous procedures have demonstrated to be useful mainly when acute or partial renal vein thrombosis occurs in the first months after renal transplantation, but, to our knowledge, this technique has never been used to treat ARVT caused by DVT extension. Finally, thrombolysis with streptokinase, urokinase, or alteplase could also theoretically be useful; however, the risk of life-threatening hemorrhage in this early postoperative period and in the presence of a large perirenal hematoma made this choice unattractive. 

There are no guidelines for treatment of early post-transplantation renal vein thrombosis, and evidence is scarce in the literature, with studies limited by a small number of cases. Surgical thrombectomy showed successful results [20, 21, 22]. As renal vein thrombosis, in the early post-transplant period is frequently caused by technical and mechanical complications [20], surgical exploration of the allograft seems to be safe and useful, sometimes with favorable long-term outcomes (Table 1). Studies also showed that aspiration venous thrombectomy after the second transplantation week is safe and effective [23]. Recently, Rerolle et al. [8] showed two cases of early ARVT successfully treated by this technique, carried out with full heparinization in the first 2 weeks after kidney transplantation. 

Thus, as reinforced by our case, the authors believe that balloon catheter and aspiration venous thrombectomy could be a good option when the principal cause of ARVT is other than technical complications. On the other hand, the success of treatment may not be immediate. In our case, the patient remained anuric for 5 days after procedure and, similarly, Ferreira et al. [24] showed the same. 

Some reports showed that the most important predictor of good prognosis in ARVT is early intervention [20, 25]. Frequently, the diagnosis is challenging, and a strong suspicion is required. Clinical signs and symptoms of renal vein thrombosis, such as allograft tenderness and swelling, hematuria, oliguria, proteinuria, allograft dysfunction, and anuria are relatively nonspecific but can be associated with renal vein thrombosis, even without typical findings on Doppler US [22]. 

The diagnosis in the present case was supported by allograft Doppler US showing an enlarged renal vein filled with hypoechogenic content, an arterial reversed diastolic flow, and absence of Doppler signal. Because of the deep vascular anastomose in the recipient’s pelvis, thrombi in the renal vein are rarely seen. Thus, the finding of a reversed or absent diastolic arterial flow, as the unique signal, is frequent. However, this finding is not pathognomonic of renal vein thrombosis, and it may also be seen in acute rejection, severe acute tubular necrosis, hematoma, and in a vascular kink [1]. 

## Conclusion 

Although early ARVT is a severe complication, it may not always lead to nephrectomy or allograft loss. Published studies have described different approaches to manage this condition with success. The balloon catheter and aspiration venous thrombectomy followed by systematic heparinization of allograft renal vein thrombosis by extension of DVT in the first week after kidney transplantation could be a good option when the cause of ARVT is other than technical complications. The efficacy of this treatment may not be immediate; thus, the authors suggest to wait and delay nephrectomy. Lastly, the authors stress the importance of an early diagnosis. Thus, because of the nonspecific presentation, a strong suspicion is required. 

## Funding 

The author(s) received no financial support for the research, authorship, and/or publication of this article. 

## Conflict of interest 

The authors have no conflicts of interest to disclose. 

**Table 1.** Treatment of transplant renal vein thrombosis, in the first 2 weeks. Literature review.

Author	Case report	Time to ARVT	Risk factors	Treatment	Treatment time	Outcome
Rerolle et al. [8]	Case 1	POD 8	Right kidney, cold ischemia time 40 hours, ISS with MMF and CsA; delayed graft function;	PT thromboaspiration followed by full heparinization;	Immediately	Restored satisfactory vein patency (20 min). SCr at 6 months was 2 mg/dL.
	Case 2	POD 12	Right kidney, cold ischemia time 30 hours 30 minutes; DGF; ISS with CsA;	PT thromboaspiration followed by full heparinization;	Immediately	Restored satisfactory vein patency (20 minutes). SCr at 6 months was 1.4 mg/dL.
Lerman et al. [21]	Case 1	ND but until POD 15	ISS with MMF, no other was reported inclusive aberrant venous anatomy	Graft explanted and flushed with preservation solution and TPA followed by clot removal (renal and iliac vein), graft reimplantation and full heparinization.	After 1 day	Early allograft dysfunction because of significant tubular damage, however, at 17 weeks after renal vein thrombectomy SCr was 1.74 mg/dL.
Hori et al. [22]	Case 1	Uncertain POD 1 – 4	BMI 33.4 kg/m^2^, IMS with MMF, hematoma	Surgical thrombectomy	POD 4	POD 10 SCr ≈ 1.5 mg/dL
Fathi et al. [20]	Case 1	POD 0	Hematoma	Surgery thrombectomy and evacuated hematoma	Immediately	Functioning graft for more than 4 years
	Case 2	POD 2	ND	Surgical thrombectomy	Immediately	Functioning graft for more than 6 months
Kawano et al. [7]	Case 1	POD 10	Cold ischemia time 24 hours 42 minutes; ISS with MMF	Surgical approach: infusion with Euro-Collins solution by RA resulting in output vein thrombi followed by systemic heparinization	Immediately	Recovered urine output and kidney function after 1 week

BMI = body mass index; CsA = cyclosporine; DGF = delayed graft function; ISS = immunosuppressive treatment; MMF = mycophenolate mofetil; ND = not defined; POD = postoperative day; PT = percutaneous transluminal; RA = renal artery; SCr = serum creatinine; TPA = tissue plasminogen activator; ARVT = transplant renal vein thrombosis.

## References

[1] El Zorkany K Bridson JM Sharma A Halawa A Transplant renal vein thrombosis. Exp Clin Transplant. 2017; 15: 123–129. 28338457 10.6002/ect.2016.0060

[2] Dimitroulis D Bokos J Zavos G Nikiteas N Karidis NP Katsaronis P Kostakis A Vascular complications in renal transplantation: a single-center experience in 1367 renal transplantations and review of the literature. Transplant Proc. 2009; 41: 1609–1614. 19545690 10.1016/j.transproceed.2009.02.077

[3] Penny MJ Nankivell BJ Disney AP Byth K Chapman JR Renal graft thrombosis. A survey of 134 consecutive cases. Transplantation. 1994; 58: 565–569. 8091483

[4] de Freitas RAP de Lima ML Mazzali M Early vascular thrombosis after kidney transplantation: can we predict patients at risk? Transplant Proc. 2017; 49: 817–820. 28457402 10.1016/j.transproceed.2017.03.004

[5] Parajuli S Lockridge JB Langewisch ED Norman DJ Kujovich JL Hypercoagulability in kidney transplant recipients. Transplantation. 2016; 100: 719–726. 26413991 10.1097/TP.0000000000000887

[6] Ponticelli C Moia M Montagnino G Renal allograft thrombosis. Nephrol Dial Transplant. 2009; 24: 1388–1393. 19182239 10.1093/ndt/gfp003

[7] Kawano PR Yamamoto HA Gerra R Garcia PD Contti MM Nga HS Takase HM Bravin AM de Andrade LGM A case report of venous thrombosis after kidney transplantation – We can save the graft? Time is the success factor. Int J Surg Case Rep. 2017; 36: 82–85. 28550788 10.1016/j.ijscr.2017.04.022 PMC5447376

[8] Rerolle JP Antoine C Raynaud A Beyssen B Julia P Duboust A Glotz D Successful endoluminal thrombo-aspiration of renal graft venous thrombosis. Transpl Int. 2000; 13: 82–86. 10743696 10.1007/s001470050014

[9] Eufrásio P Parada B Moreira P Nunes P Bollini S Figueiredo A Mota A Surgical complications in 2000 renal transplants. Transplant Proc. 2011; 43: 142–144. 21335172 10.1016/j.transproceed.2010.12.009

[10] Ramirez PJ Gohh RY Kestin A Monaco AP Morrissey PE Renal allograft loss due to proximal extension of ileofemoral deep venous thrombosis. Clin Transplant. 2002; 16: 310–313. 12099990 10.1034/j.1399-0012.2002.02006.x

[11] Goldman MH Leapman SB Handy RD Best DW Renal allograft rupture with iliofemoral thrombophlebitis. Arch Surg. 1978; 113: 204–205. 343749 10.1001/archsurg.1978.01370140094021

[12] Cho J Jun KW Kim MH Hwang JK Moon IS Kim JI Coagulation profile in patients with chronic kidney disease before and after kidney transplantation: A retrospective cohort study. Clin Transplant. 2017; 31: e13051. 10.1111/ctr.13051 28678346

[13] Opatrný K Zemanová P Opatrná S Vít L Fibrinolysis in chronic renal failure, dialysis and renal transplantation. Ann Transplant. 2002; 7: 34–43. 12221902

[14] Ghisdal L Broeders N Wissing KM Mena JM Lemy A Wijns W Pradier O Donckier V Racapé J Vereerstraeten P Abramowicz D Thrombophilic factors in Stage V chronic kidney disease patients are largely corrected by renal transplantation. Nephrol Dial Transplant. 2011; 26: 2700–2705. 21285127 10.1093/ndt/gfq791

[15] Allen RD Michie CA Murie JA Morris PJ Deep venous thrombosis after renal transplantation. Surg Gynecol Obstet. 1987; 164: 137–142. 3544275

[16] Alkhunaizi AMO Olyaei AJ Barry JM deMattos AM Conlin MJ Lemmers MJ Bennett WM Norman DJ Efficacy and safety of low molecular weight heparin in renal transplantation. Transplantation. 1998; 66: 533–534. 9734500 10.1097/00007890-199808270-00020

[17] Robertson AJ Nargund V Gray DW Morris PJ Low dose aspirin as prophylaxis against renal-vein thrombosis in renal-transplant recipients. Nephrol Dial Transplant. 2000; 15: 1865–1868. 11071979 10.1093/ndt/15.11.1865

[18] Stechman MJ Charlwood N Gray DW Handa A Administration of 75 mg of aspirin daily for 28 days is sufficient prophylaxis against renal transplant vein thrombosis. Phlebology. 2007; 22: 83–85. 18268856 10.1258/026835507780346187

[19] Mathis AS Davé N Shah NK Friedman GS Bleeding and thrombosis in high-risk renal transplantation candidates using heparin. Ann Pharmacother. 2004; 38: 537–543. 14766999 10.1345/aph.1D510

[20] Fathi T Samhan M Gawish A Donia F Al-Mousawi M Renal allograft venous thrombosis is salvageable. Transplant Proc. 2007; 39: 1120–1121. 17524908 10.1016/j.transproceed.2007.03.043

[21] Lerman M Mulloy M Gooden C Khan S Khalil A Patel L Zhou XJ Post transplant renal vein thrombosis, with successful thrombectomy and review of the literature. Int J Surg Case Rep. 2019; 61: 291–293. 31401437 10.1016/j.ijscr.2019.07.066 PMC6699557

[22] Hori S Miyamoto T Sakamoto K Shimizu T Ichikawa K Morizawa Y Gotoh D Nakai Y Miyake M Yoneda T Tanaka N Yoshida K Fujimoto K Successful salvage of allograft dysfunction triggered by transplant renal vein thrombosis immediately after kidney transplantation: a case report. Int J Nephrol Renovasc Dis. 2018; 11: 321–327. 30538528 10.2147/IJNRD.S185520 PMC6260141

[23] Melamed ML Kim HS Jaar BG Molmenti E Atta MG Samaniego MD Combined percutaneous mechanical and chemical thrombectomy for renal vein thrombosis in kidney transplant recipients. Am J Transplant. 2005; 5: 621–626. 15707419 10.1111/j.1600-6143.2004.00696.x

[24] Ferreira C Pereira L Pereira P Tavares I Sampaio S Bustorff M Pestana M Late allograft renal vein thrombosis treated with anticoagulation alone: A case report. Transplant Proc. 2016; 48: 3095–3098. 27932155 10.1016/j.transproceed.2016.09.006

[25] Harraz AM Shokeir AA Soliman SA Osman Y El-Hefnawy AS Zahran MH Kamal AI Kamal MM Ali-El-Dein B Salvage of grafts with vascular thrombosis during live donor renal allotransplantation: a critical analysis of successful outcome. Int J Urol. 2014; 21: 999–1004. 24861882 10.1111/iju.12485

